# The Bayesian Decoding of Force Stimuli from Slowly Adapting Type I Fibers in Humans

**DOI:** 10.1371/journal.pone.0153366

**Published:** 2016-04-14

**Authors:** Patrick Kasi, James Wright, Heba Khamis, Ingvars Birznieks, André van Schaik

**Affiliations:** 1 The MARCS Institute for Brain, Behaviour and Development, Western Sydney University, Sydney, NSW, Australia; 2 Graduate School of Biomedical Engineering, University of New South Wales, Sydney, NSW, Australia; 3 School of Medical Sciences, University of New South Wales, Sydney, NSW, Australia; 4 Neuroscience Research Australia, Sydney, NSW, Australia; McGill University, CANADA

## Abstract

It is well known that signals encoded by mechanoreceptors facilitate precise object manipulation in humans. It is therefore of interest to study signals encoded by the mechanoreceptors because this will contribute further towards the understanding of fundamental sensory mechanisms that are responsible for coordinating force components during object manipulation. From a practical point of view, this may suggest strategies for designing sensory-controlled biomedical devices and robotic manipulators. We use a two-stage nonlinear decoding paradigm to reconstruct the force stimulus given signals from slowly adapting type one (SA-I) tactile afferents. First, we describe a nonhomogeneous Poisson encoding model which is a function of the force stimulus and the force’s rate of change. In the decoding phase, we use a recursive nonlinear Bayesian filter to reconstruct the force profile, given the SA-I spike patterns and parameters described by the encoding model. Under the current encoding model, the mode ratio of force to its derivative is: 1.26 to 1.02. This indicates that the force derivative contributes significantly to the rate of change to the SA-I afferent spike modulation. Furthermore, using recursive Bayesian decoding algorithms is advantageous because it can incorporate past and current information in order to make predictions—consistent with neural systems—with little computational resources. This makes it suitable for interfacing with prostheses.

## Introduction

It has been demonstrated that tactile afferents—associated with cutaneous mechanoreceptors—signal information to the brain that is relevant for dexterous object manipulation in humans [1, 2]. While each of the four types of afferents [3–6] has unique properties, they may encode the same stimulus features jointly [7–12]. Fast adapting type I (FA-I) afferents respond to dynamic skin deformations of relatively high frequencies (5–60 ms), slowly adapting type I (SA-I) afferents are tuned towards low frequency skin deformations of the glabrous skin [13, 14], fast adapting type II (FA-II) afferents are optimized for detecting transient mechanical events [13–16], and slowly adapting type II (SA-II) afferents respond to remotely applied lateral stretching of the skin [3, 17]. However, a complete understanding of the mechanisms that underlie information signaling remains elusive: As an example, the contribution of SA-I afferents towards the encoding of force is yet to be assessed quantitatively [18]. In order to gain insight into the representation and consequent reconstruction of properties of the object and motor control, a systematic approach within a quantitative framework that is simple to interpret is of interest. This will facilitate studying factors that concurrently contribute to the afferent spiking behavior. Kim et al. [19] used an intricate integrate and fire model—driven by a linear combination of indentation depth and its higher derivatives—to encode and simulate tactile afferent data under various stimuli. Neural decoding techniques that avoid explicit encoding models (reverse correlation) have been applied [20–22], and recently extended to tactile afferent data [23].

Another way of estimating continuous values given neural spike data (neural decoding) is by implementing a dual paradigm—encoding and decoding, respectively—within a Bayesian framework [24–33]. While the encoding stage involves a probabilistic mapping of the relationship between the recorded afferent spike data and the stimulus that led to the afferent spike response, the decoding stage aims to reconstruct the most likely values of the stimulus given the afferent spike data. The Bayesian decoding framework offers a more flexible means of analysis—unlike regression methods [34]. For instance, it is possible to do statistical inferences [27, 35], and also possible to capture nonlinear relationships between the stimulus and the corresponding neural spikes [27, 34, 36]. A dual paradigm based on Bayesian methods is yet to be extended to the analyses of tactile afferent data. First, a parametric statistical model is used to capture the relationship (dependence) between the tactile afferent spiking data and the force stimuli and its higher order derivatives. In this way, we can assess the relative importance of the higher order derivatives of the stimulus on the afferents’ propensity to spike at some time *t*. Furthermore, methodologies essential to the reconstruction of the continuous force stimuli given the spike data, are described. A second stage implements a recursive algorithm to estimate continuous values. The estimated continuous values represent the stimulus. Implementing these methods should yield improved quantitative descriptions of how tactile afferents represent information about properties between the glabrous skin of the hand and objects.

To apply these methods, we use SA-I afferent data to reconstruct the corresponding force stimulus. The SA-I afferent spike data were elicited, and recorded at the median nerve, during the application of a force stimulus at the tip of the human finger-pad. The parameters estimated from the model fit to the data can capture relationships between afferent spike activity and the covariates. As a result, statistical hypothesis tests can be used to quantitatively assess the relative importance of model components. In addition, through goodness-of-fit analyses, we can identify afferent spike data properties that the model cannot capture. A description of the mapping of afferent spike trains into a continuous signal would demonstrate a possible way of how the central nervous system interprets and converts spike train information into signal predictions. In an on-line setting, decoding will be implemented based on current and previous inputs, a technique in agreement with the sequential way neural systems update—the current signal prediction is computed from the previous signal prediction plus the new information in the spike train about the change in the signal since the previous prediction. In the encoding stage we model SA-I spiking activity as a nonhomogeneous Poisson process whose instantaneous firing rate is a function of the force indenting the tip of the finger-pad and its higher order derivatives. In the decoding stage we use Bayesian statistical theory to derive a nonlinear, recursive filter algorithm for reconstructing the force stimulus from a population of SA-I afferent spike patterns.

## Methods

### Data

Eight subjects, 24.3 ± 5.7 years of age (mean ± SD) participated in this study. The study was approved by the local ethics committee, at Western Sydney University (Ethics Approval H9967). The procedures followed were in accordance with the ethical standards of the local ethics committee on human experimentation and with the Helsinki Declaration of 1975, as revised in 2000. Each participant had no known neurological disorders. All subjects provided written informed consent before taking part in the study. Normal force was applied to the finger-pad of an immobilised finger of the right hand using a six axis robotic manipulator AGILUS R900 (KUKA Roboter GmbH, Germany). A force transducer (Nano F/T, ATI Industrial Automation, Garner, USA) was attached at the tip of the robotic manipulator. The robot was programmed to safely deliver the force stimulus at the human finger-tip. Upon touching the finger-tip, the robotic manipulator switched from position to force control mode. A device to immobilize the finger was used. The device was adjustable and could accommodate different finger sizes.

Tactile data were recorded from SA-I afferent fibers of the right hand. The needle electrode was percutaneously inserted into the median nerve and positioned in such a way as to obtain action potentials (AP) waveforms [37, 38]. Force profiles and the corresponding tactile afferent signals were recorded simultaneously, using a 16-bit data acquisition system (PowerLab, ADInstruments; Dunedin New Zealand). Force data were sampled at 1kHz and afferent data were sampled at 20kHz. The acquisition system was set up with a monitor to provide visual feedback, and speakers to provide audio feedback. The feedback from the monitor and speakers was used to ensure that the quality of the data recorded is suitable for analysis. Spike sorting techniques—where the occurrences of AP waveforms that pertain to an individual cell are grouped—were applied to the afferent data based on methods described in [39–41]. In cases where AP waveforms overlapped, as result of recording from more than one afferent fiber, we used a combination of automated and visual methods to identify which afferent fibers contributed to that AP waveform.

**Fig 1:**
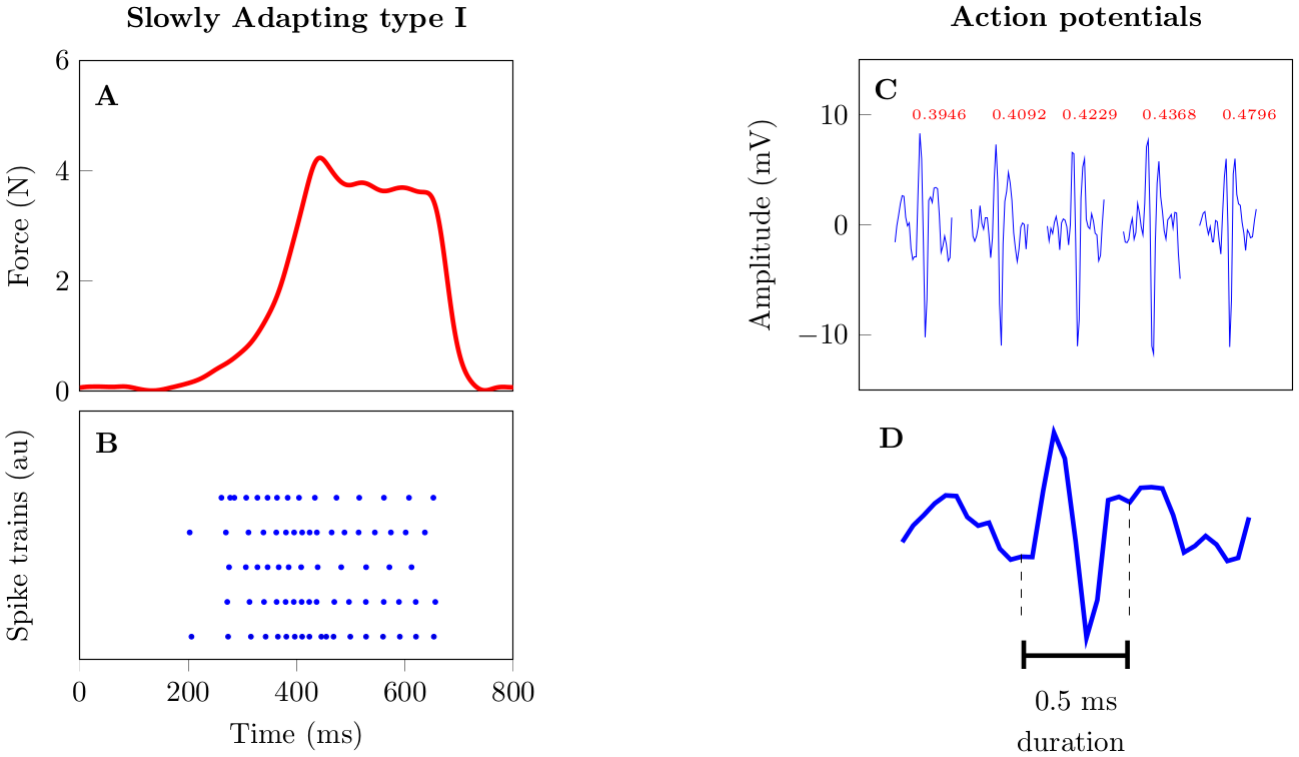
Example of SA-I firing characteristics. Panel **A** shows the stimulus used to elicit slowly adapting type I tactile response shown in **B**. Panel **C** shows a representation of a series of action potentials along with their corresponding times of occurrence (in ms). A zoomed in version of the left most action potential is represented in panel **D**. We fit a nonhomogeneous Poisson model to the first portion of the data (100–450 ms). The inverse problem—decoding—was done using data in the range 451–750 ms.

Fig 1 shows an example of a force profile and the corresponding (spike sorted) neural spikes that were recorded from an SA-I afferent (Panels **A** and **B** respectively). We apply the methods described below, to an ensemble of 28 SA-I afferents.

### Statistical methods

SA-I afferents are associated with Merkel discs that encode information about some properties of the object in the hand into neural spike patterns. We devise a model (encoding) to capture the mapping between the force stimulus and the corresponding SA-I afferent spike response. The data were split into two disjoint subsets. A subset was used to fit a model (encoding) and another was used to assess how well the decoding algorithms generalize. The encoding subset was defined as the data recorded during the first portion of recording (between 100–450 ms, see Fig 1). This subset was used to fit the nonhomogeneous Poisson process model for each SA-I afferent. The second subset was defined as the data recorded during the rest of the recording period (between 451–750 ms) and was used to reconstruct the force stimulus using a recursive Bayesian filter.

#### Encoding model

We define the model for SA-I afferents using a nonhomogeneous Poisson process. A nonhomogeneous Poisson process is a Poisson process where the rate parameter varies as a function of time and/or some other physical quantity but it retains the memoryless property [42]. In this study, the rate parameter of the nonhomogeneous Poisson process is modeled as a function of the force stimulus and the derivative of the force stimulus. This is because among three candidate models—a first where we consider force only, a second where we take a combination of force and its derivative, and a third where force as well as its first and second derivatives are considered. We used the model which considers the force and its first derivative because this model yielded the lowest Akaike’s Information Criteria (AIC) value [43]—for each of the afferents under the current model. The encoding model is defined as follows:

$$\begin{array}{c} \lambda \left(t|s\left(t\right),\beta_{0},\beta \right)=\exp\left(\beta_{0}+\beta S\left(t\right)\right) \end{array}$$ (1)

where *β*_0_ corresponds to the baseline firing rate, ***β*** is the vector of parameters corresponding to covariates that modulate firing rate, and **S**(*t*) is a matrix of covariates that modulate the firing activity. We assume that individual SA-I afferents form a population of conditionally independent Poisson processes (the SA-I afferents are independent given their model parameters). We fit the nonhomogeneous Poisson model defined in Eq 1 to each SA-I afferent. We estimated the model parameters based on the maximum likelihood method [44, 45]. The relative importance of the first and second derivatives of the components were assessed using Akaike Information Criterion (AIC) [27, 46].

#### Assessment of goodness-of-fit

After fitting the model to data, we assessed its validity in describing the observed SA-I afferent spike data. In order to use already established statistical methods, such as the Kolmogorov-Smirnov (K-S) test, we transformed the data into a simpler form. The *Time rescaling* theorem, in addition to simulation of point process data, can be used to transform the data [47–49]. Using the rate (conditional intensity function), estimated from the data, we transformed the data using time rescaling to obtain a homogeneous Poisson process with rate equal to one, and further transformed the data into uniform random variables:

$$\begin{array}{c} u_{j}=1-\exp\left(\int_{t_{j-1}}^{t_{j}}\lambda_{a}\left(t|s\left(t\right),\beta_{0a},\beta_{a}\right)\right)\;dt \end{array}$$ (2)

where *t*_*j*_ is the spike time, *u*_*j*_ is a uniform random variable. We then use the K-S test to assess how close the empirical distribution of rescaled spike times are to a reference uniform distribution on the interval (0, 1). If the nonhomogeneous model described fit the data correctly, the transformed data should lie on a 45° line on the K-S plot.

#### Decoding model

The state transition function for the force is defined as follows:

$$\begin{array}{c} s_{t_{l}}-s_{t_{l-1}}=\dot{s}\Delta_{t_{l-1}} \end{array}$$ (3)

Following the encoding stage, the decoding stage aims to find the best estimate of *s*(*t*_*l*_) for each *t*_*l*_ using a probability density given the A afferents, force and force derivative parameters. To facilitate the description of the decoding procedure, we start by defining a set of times in (*t_ϱ_*, *T*], *t_ϱ_* ≤ *t_0_* < *t_1_* <, ⋯, < *t_l_* < *t_l+1_*, ⋯, *t_L_* ≤ T, and let *ΔN*_*a*_(*t*_*l*_) be an indicator function. The indicator function is equal to one if there is a spike at time *t*_*l*_ and zero if there is no spike at time *t*_*l*_. We let $\Delta N\left(t_{l}\right)={\left[\Delta N_{1}\left(t_{l}\right),\cdots ,\Delta N_{A}\left(t_{l}\right)\right]}^{⊺}$ be a vector of all A afferents at time *t*_*l*_. The probability density of *s*(*t*_*l*_), given the spikes in (*t_ϱ_*, *T*]) and parameters estimated during the encoding stage, is computed sequentially using Bayes’ rule from probability densities of previous force and force derivatives and that of the new afferent data recorded since the previous state prediction was made [27], [50]. The formulation of the recursive algorithm is based on two steps: the prediction and the update. The prediction stage is based on the relationship between the posterior, at the previous time step, and the state evolution model. The one-step prediction probability density is defined below.

$$\begin{array}{c} Pr\left(s\left(t_{l}\right)|\Delta N_{t_{\varrho }:t_{l-1}}\right)=\int ds\left(t_{l-1}\right)\;Pr\left(s\left(t_{l-1}\right)|\Delta N_{t_{\varrho }:t_{l}}\right)Pr\left(s\left(t_{l}\right)|s\left(t_{l-1}\right)\right) \end{array}$$ (4)

The equations for tracking the mean and variance of the one-step prediction are defined below:

$$\begin{array}{c} \bar{s}\left(t_{l}|t_{l-1}\right)=F\bar{s}\left(t_{l-1}|t_{l-1}\right) \end{array}$$ (5)

$$\begin{array}{c} W\left(t_{l}|t_{l-1}\right)=FW\left(t_{t-1|t-}\right)F^{⊺}+Q \end{array}$$ (6)

where *F* is the state transition matrix, and Q is the covariance matrix of a Gaussian process with zero mean. *F* was assumed to be a linear evolution system because we observed that neighboring force values are very close to one another.

In the update stage, we use Bayes rule to get the posterior probability density and is defined as follows:

$$\begin{array}{c} \frac{Pr\left(s_{t_{l}}|\Delta N_{t_{\varrho }:t_{l-1}}\right)Pr\left(\Delta N_{t_{l}}|s(t_{l},\Delta N_{t_{\varrho }:t_{l-1}}\right))}{Pr\left(\Delta N_{t_{l}}|\Delta N_{t_{\varrho }:t_{l-1}}\right)} \end{array}$$ (7)

where the second part in the numerator of Eq 7 is the likelihood of observing a spike. The likelihood function of A afferents is described below,

$$\begin{array}{c} p\left(\Delta N\left(t_{l}\right)|s\left(t_{l}\right),\Delta N_{t_{\varrho }:t_{l-1}}\right)=\prod_{a}^{A}{\left[\lambda_{a}\left(t_{l}\right)\right]}^{\left(\Delta N_{a}\left(t_{l}\right)\right)}\exp\left(-\left[\lambda_{a}\left(t_{l}\right)\right]\right) \end{array}$$ (8)

We track the posterior mean and posterior variance as follows:

$$\begin{array}{c} \bar{s}\left(t_{l}|t_{l}\right)=\bar{s}\left(t_{l}|t_{l-1}\right)+W_{t_{l}|t_{l}}\sum_{a=1}^{A}\left[{\left(\frac{\partial \log\lambda (t_{l})}{\partial s(t_{l})}\right)}^{⊺}\left(\Delta N_{a}\left(t_{l}\right)-\lambda_{a}\left(t_{l}\right)\right)\right] \end{array}$$ (9)

where

$$\begin{array}{ccc} W_{t_{l}|t_{l}}^{-1} & = & W_{t_{l}|t_{l-1}}^{-1}+\sum_{a=1}^{A}\\ & & [{\left(\frac{\partial \log\lambda_{a}\left(t_{l}\right)}{\partial s(t_{l})}\right)}^{⊺}\lambda_{a}\left(t_{l}\right)\left(\frac{\partial \log\lambda_{a}\left(t_{l}\right)}{\partial s(t_{l})}\right)\\ & & -\left(\Delta N_{a}\left(t_{l}\right)-\lambda_{a}\left(t_{l}\right)\right)\frac{\partial^{2}log\lambda_{a}\left(t_{l}\right)}{\partial s\left(t_{l}\right)\partial {\left(s\left(t_{l}\right)\right)}^{⊺}}{]}_{t_{l}|t_{l-1}} \end{array}$$ (10)

## Results

Our main finding is that for slowly adapting type I tactile afferents, the force derivative (in addition to the force) is an external factor that influences spike behavior. As a consequence, it is an essential component when studying relationships between observed spikes and a force stimulus. Furthermore, we can predict force profiles given results based on parameters learned during the encoding model and slowly adapting type I tactile afferents. More details about these findings are presented in following sections.

### Encoding

We used a nonhomogeneous Poisson model, described in the methodology section, to fit to the SA-I tactile afferent spike data. SA-I afferent spike data with negative force coefficients (*β*_1_) were removed. There were seven such spike trains, and they were removed because the quality of the recording may not have been good for reliable identification of spikes. For each of the 28 SA-I afferents, the firing rate was highest in the region with highest force and highest rate of change of the force stimulus. The inclusion of force derivative, based on Akaike Information Criteria (AIC), resulted in an improvement (lower AIC value) in the fit of the model for 26 of the 28 afferents. A Wilcoxon Signed-Ranks Test indicated that AIC values for the model that considers force only was statistically significantly higher than AIC values of the model considering force and its first derivative (*p* < 0.001, significance level *α* = 0.05, two tailed). We also compared AIC values of the model that accounted for force, its first and second derivatives, against the model that considers just force and its derivative. A Wilcoxon Signed-Ranks Test indicated that AIC values for the model that considers force, and its first and second derivatives was statistically significantly higher than AIC values of model considering force and its derivative (*p* < 0.001, significance level *α* = 0.05, two tailed). We selected the model that considers force and its first derivative. The force and force derivative modulation components of the nonhomogeneous Poisson model are consistent with previous studies; that is, the firing propensity increases with increasing force and the first derivative of the force [6].

**Fig 2:**
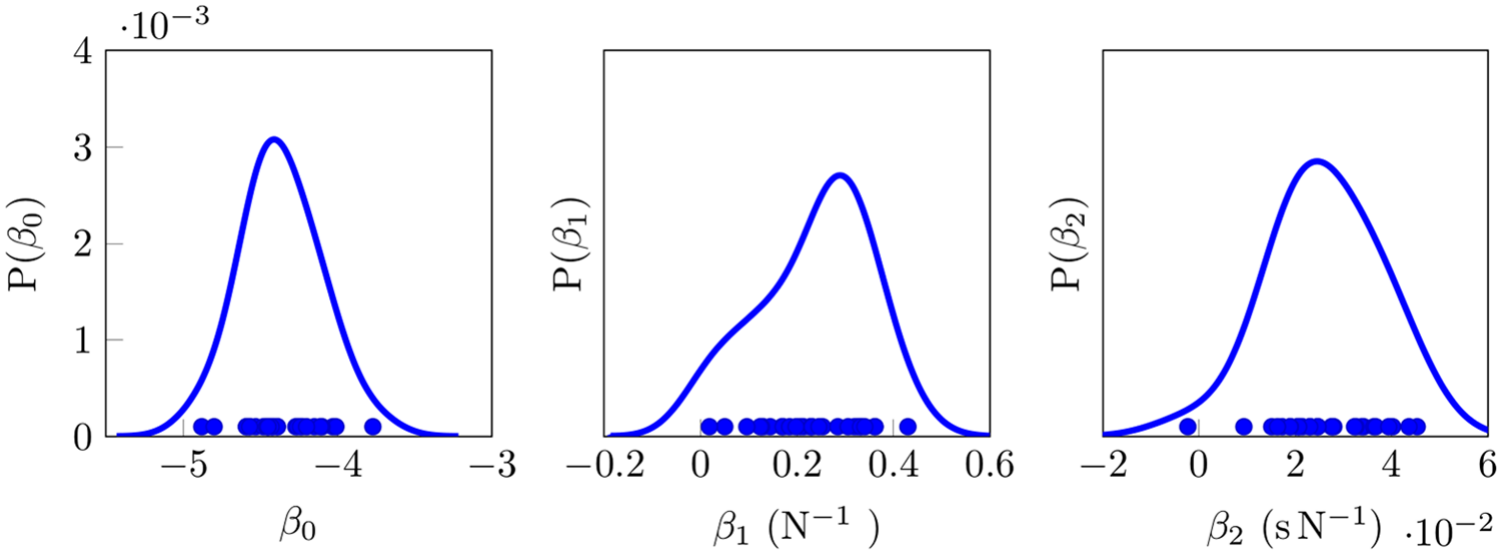
Distribution of parameters estimated from the data based on the nonhomogeneous Poisson model. In this Figure, parameter *α* corresponds to the baseline firing rate, parameter *β*_1_ corresponds to the force stimulus, and parameter *β*_2_ corresponds to the rate at which the force changes. The dots represent the actual parameter estimates from individual SA-I afferents.

Parameters were estimated individually for each SA-I afferent, and are distributed as shown Fig 2. Estimating parameters individually allows for the direct quantitative assessment of the relative importance of force and force derivative on SA-I firing. To illustrate, we use parameters estimated using the SA-I afferent shown in Fig 2: we take the force (*f*) and force derivative (*f*′) values at *t* = 400ms (2.89N and 37.38Ns^−1^) and estimate the spike rate. The estimated spike rate under nonhomogeneous model that considers force only is, exp(*β*_0_ + *β*_1_ *f*) = exp(−4.26 + 0.45 × 2.89), ≈52 spikes per second. However when the force derivative is taken into account the rate is estimated to be, exp(*β*_0_ + *β*_1_ *f* + *β*_2_ *f*′) = exp(−4.42 + 0.33 × 2.89 + 0.02 × 37.38), ≈ 69 spikes per second. Because we have a relatively small number of SA-I afferents, we use the median to assess their central tendency [27]. The median of the estimated parameters is: *β*_0_ = −4.42, *β*_1_ = 0.23, and *β*_2_ = 0.02. The median ratio of the force to the derivative of the force is: exp(0.23) = 1.26 to exp(0.02) = 1.02. These results suggest that the force derivative, in addition to the force, contributes to the modulation of SA-I firing rate under the proposed model.

#### Assessment of model fit

**Fig 3:**
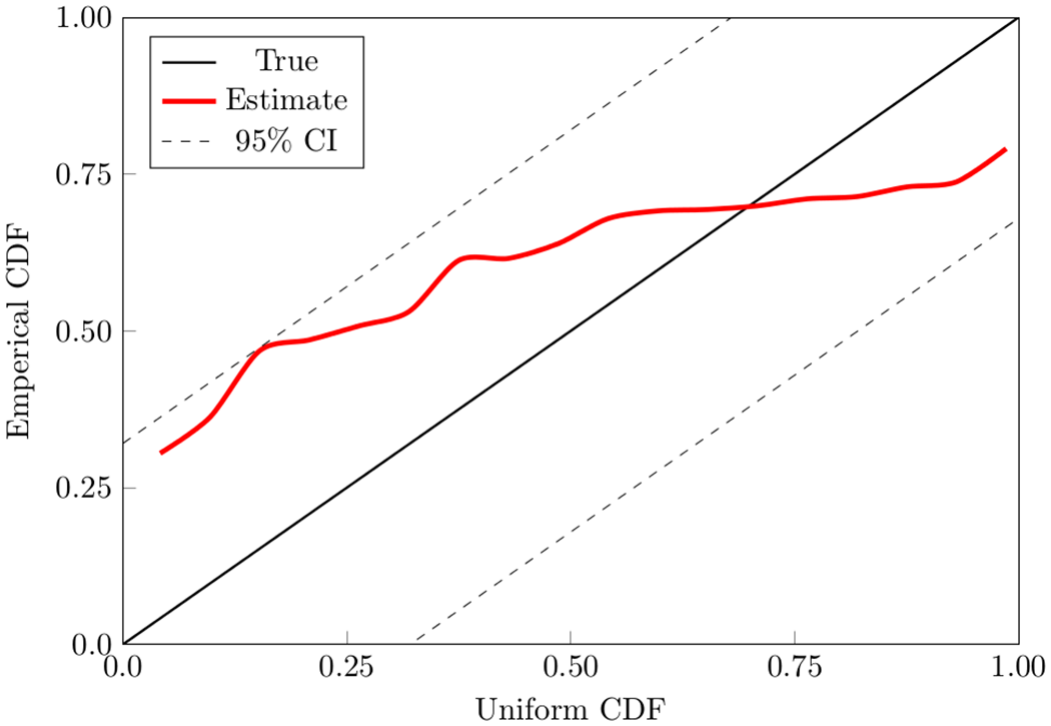
Goodness-of-fit assessment (K-S plot) of proposed model. If the model describes the data well, the estimated distribution should follow a forty-five degree line. The 95% confidence intervals for the Kolmogorov-Smirnov statistic are computed by um±1.36(n2), where $u_{m}=\left(m-\frac{1}{2}\right)/n$ are the values of the cumulative distribution (CDF) of a uniform random variable, *m* = 1, 2, ⋯, *n*, and *n* is the number of interspike intervals. Results show that the Poisson model does not describe the data well.

We used time rescaling to assess model goodness-of-fit. Time rescaling transforms the rate into identically distributed exponential random variables with mean rate one. A further transformation is done to obtain uniform random variables in the interval (0, 1). Based on the transformed data, we use the K-S test [48]. Results show that the model captures properties of the data reasonably well as shown in Fig 3. While the model is useful in describing some aspects of SA-I spiking behavior, it does not account for a number factors that contribute to the observed spiking behavior. Implementing a model that accounts for other covariates and spike history may lead to improved decoding of SA-I afferents.

### Decoding

Due to the limited number of trials, we pooled data from individual trials to form a population of spike trains [51–54], and then decoded the average force profile. The 28 SA-I afferents were pooled from across multiple trials by considering the interval: 100ms before stimulus onset and 100ms after stimulus offset, see Fig 1.

**Fig 4:**
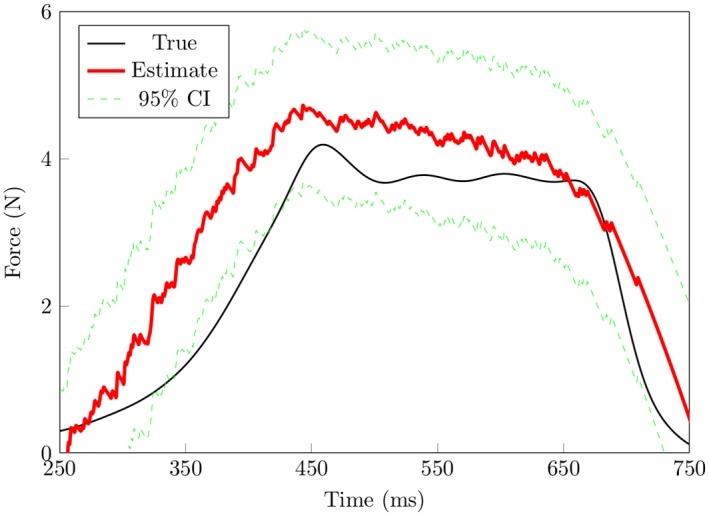
Decoding of entire force profile. We split the data into two sets of equal number of afferents. We used the first half of the data to encode and the other half to decode. Using this we have less spike trains for the decoding operation and may explain the relatively poor performance.

**Fig 5:**
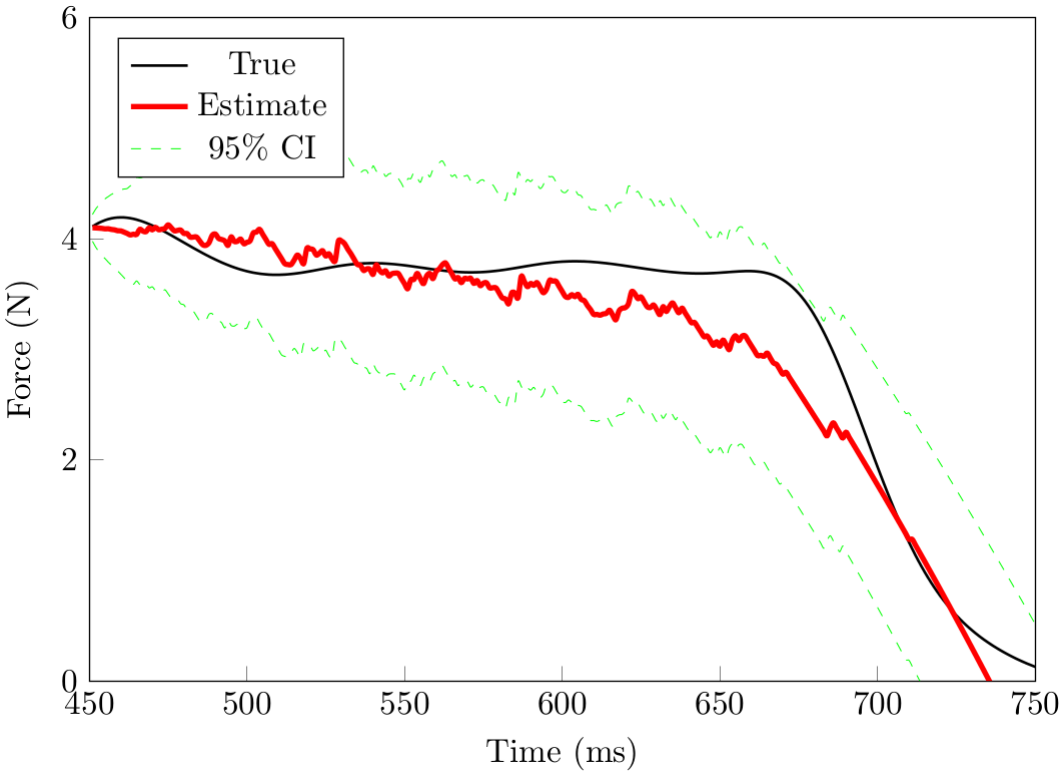
Recursive decoding results based on subset of the data not seen by the encoding model. In this scheme, the first portion of the data, as described in the Methods section, was used to map the relationship between the force profiles and the corresponding SA-I afferent spike activity (encoding). Then using parameters estimated from the encoding stage and the rest of the SA-I afferent data, the force stimulus is predicted. Here we use all 28 SA-I afferents to decode and results show that the algorithms generalize well. The performance of the filter is less accurate during the off-loading phase (period just before contact at the finger-pad is lost) when compared to that during the plateau phase. It is likely because SA-I afferents do not respond during this period (and at point just when contact is made). The model, for example at the on-loading phase may not have sufficient information due to latency. It is also possible that decoding would improve if we consider other types of afferents like the FA-II because they are the most sensitive.

**Fig 6:**
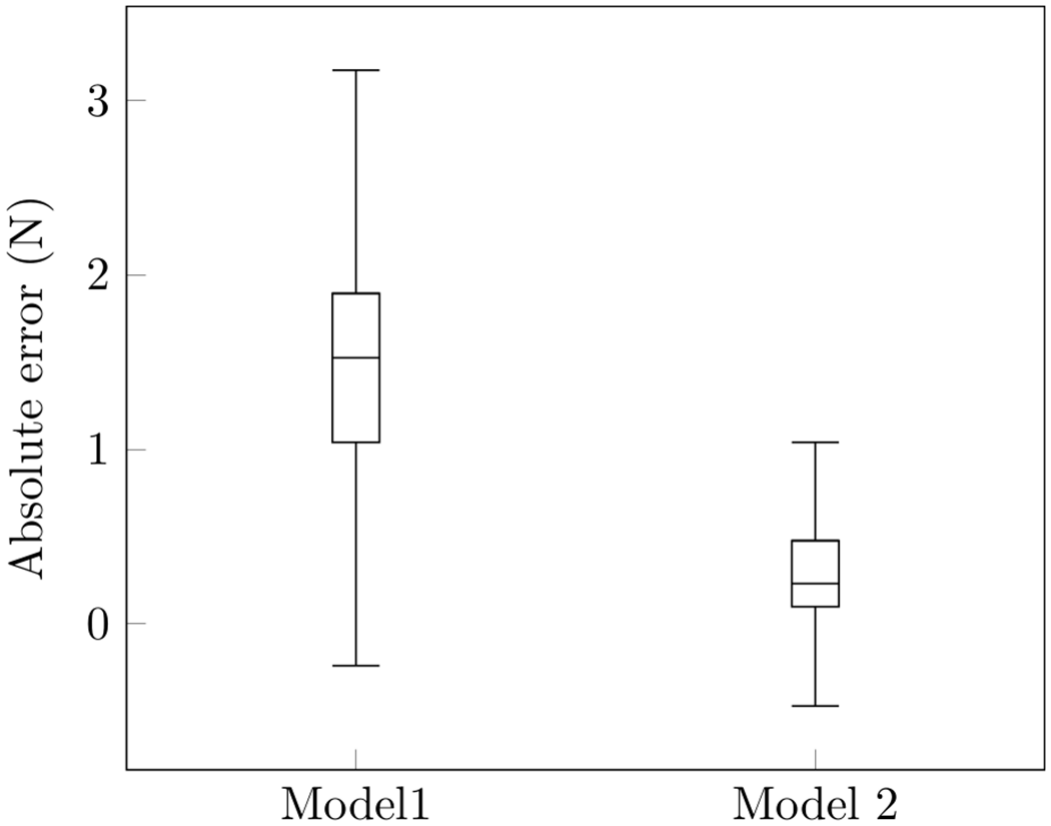
Comparison of performance between model that accounts for force only and model that accounts for force and the derivative of the force. In order to compare our model against a model that simply accounts for the force, we take the absolute difference between the true values and the predicted values for each model. Under this framework, the model that accounts for force derivative, in addition to force, performs better.

Figs 4 and 5 show results of the force reconstruction. For results based on Fig 4, spike data were split into half. One half was used for training the other half was used to assess how well the model performs. One disadvantage with this approach is that we are left with fewer spike trains for decoding. Fig 5 shows results based on a recursive Bayes’ filter, given signals from 28 SA-I afferents. The decoding of smooth force trajectories is fairly accurate. The estimation at the points of loading and unloading is spurious. This is likely because there is no SA-I afferent responses (these are points of very low forces). It is not surprising that the model did make reasonable predictions, and may suggest that FA-I afferent signals carry information associated with the points of loading and unloading. We also estimated force profiles based on an encoding model that only considered force (left out the force derivative). Results based on Fig 6 show that decoding results based on this model are worse than the model that considers both the force and the first derivative of the force, at all phases of the force profile. Overall, the recursive algorithm, based on the encoding model that considers both the force and the derivative of the force performed well in predicting the force stimulus.

## Discussion

As a first step, the nonhomogeneous Poisson model we used gives a reasonable approximation to the SA-I afferent spike data as a function of the force and the derivative of the force. The model describes each SA-I afferent spike train data using three parameters: baseline firing, force stimulus, and the derivative of the force. The model allows for quantitative assessment of the relative importance of the derivative and its higher order derivatives of the spike patterns observed in SA-I afferents. Based on our results, as shown in Fig 5, good predictions of the force stimulus can be made from a population of 28 SA-I afferents. These results suggest that SA-I afferents carry a substantial amount of information about the force stimulus and its first derivative and, in addition, that this information can be quantitatively captured using a nonhomogeneous Poisson model. These results extend decoding work of Ruiz et al. [55], Aimonetti et al. [56], and Khamis et al. [23]. Ruiz et al. [55] used a population vector algorithm to study how tactile stimuli is represented in the motor cortex. Aimonetti et al. [56], implemented a population vector algorithm to predict direction of limb movements via cutaneous afferents. Khamis et al. [23] used a multiple linear regression algorithm to study force and torque prediction from populations of SA-I and FA-I afferent firing patterns recorded in monkeys respectively. They reported that the force stimulus can be predicted from a population—58—of the SA-I afferent type alone. This result agrees with our findings: We predicted force stimulus from 28 SA-I afferents recorded in humans. Nonlinear decoding results, based on Bayesian filters, show that the force stimulus representation can be updated, sequentially based on the spiking activity of the SA-I afferents.

### Encoding model

Our encoding model differs from that by Kim et al. [18] in that it summarizes the data with far fewer parameters (three), identifies stimulus components that are relevant for spike modulation (force stimulus and its derivative), and allows for the goodness-of-fit assessment. The goodness-of-fit assessment is an important aspect of our approach, and this is because it can reveal properties of the data not captured by the model. This, in turn, guides us in proposing strategies for refining the model. Although the nonhomogeneous Poisson model is a good starting point for the encoding of SA-I afferents, it is limited in that it inherently assumes that the instantaneous rate and variance of the firing rate are equal and that there is no spike history dependence [42].

### Decoding

The recursive Bayesian methods we implemented provide good force prediction results. Our decoding implementation differs from that of Ruiz et al. [55], Aimonetti et al. [56], Khamis et al. [23] in that the continuous signal values (force and force derivative), at the current time, are estimated by incorporating information from the new afferent spike data since the previous estimate, the previous signal value estimates, and the likelihood function of spike data. This approach is in agreement with the way neural systems update and predict. Furthermore the methods implemented here are nonlinear, in agreement with findings that the properties of tactile objects undergo a nonlinear transformation at the periphery [57–59]. As shown in Fig 5, the decoding algorithm predicts the force profile well. There is a larger deviation of the prediction of the force profile during force retraction, when compared to the plateau region of the force profile. This suggests that other afferent types may be needed. For example, FA-II afferents (the most sensitive afferent type with lowest thresholds) may indeed encode information about the moment of contact and the moment force stimulus contact ends, and that including them in the decoding procedure would yield improved results.

## Conclusion

We have analyzed SA-I afferent data using methods that are useful for providing insight into quantifying how populations of SA-I afferents spiking patterns encode information about the force stimulus. This methodology is also useful for identifying the relevant covariates that contribute to the neural spike patterns and for suggesting mechanisms underlying encoding.

The two major steps in our analysis paradigm are; the representation of the relation between the population spiking activity given the signal with a parametric statistical model, and the recursive application of Bayes’ theorem to predict the signal (force stimulus) from the population SA-I afferent spiking activity. The information content of the spike train is quantified in terms of the force signal predictions. The advantage of the current paradigm is in its ability to incorporate past and current information in order to make predictions—consistent with neural systems—and with little computational resources, making it suitable for interfacing with prostheses [30, 60].

While linear regression based decoders may be effective, they do not treat firing rates as stochastic but known constants. In contradistinction, the Bayesian approach models the spike trains as a stochastic point process and the force stimulus as a stochastic process based on known or reasonably assumed properties. Bayesian decoders have also been shown to be more accurate when compared to linear decoders [27, 34, 61, 62]. Although our implementation may be limited because we make Gaussian assumptions of the posterior density, Bayesian decoders are optimal in the sense that signal estimates have the smallest errors. Future work should consider a Bayesian decoders based on all types of tactile afferent signals. This may lead to improved results. Bayesian decoding techniques based on particle filtering would provide better results when compared to point process filter however, they are computationally burdensome and therefore are not suitable for real-time neural decoding which is essential when controlling neuroprothetic devices [32].
